# Research as usual in humanitarian settings? Equalising power in academic-NGO research partnerships through co-production

**DOI:** 10.1186/s13031-021-00399-w

**Published:** 2021-08-26

**Authors:** Michelle Lokot, Caitlin Wake

**Affiliations:** Health Services Research & Policy, London School of Hygiene & Tropical Medicine, 15-17 Tavistock Place, London, WC1H 9SH UK

**Keywords:** Co-production, Humanitarian, Research

## Abstract

**Background:**

Research partnerships in conflict-affected and humanitarian settings can reveal complex power hierarchies between academics and NGOs. During the process of research, decision-making may skew in favour of more powerful actors, who often direct the scope of the research, hold the budget and lead the analysis. Co-production is increasingly emerging as a helpful approach that attempts to equalise power dynamics during research. The aim of this paper is to draw attention to the main challenges associated with a “research as usual” approach to research partnerships in humanitarian settings, as power hierarchies may be particularly magnified in these settings.

**Methods:**

This paper is based on a comprehensive literature review and 32 semi-structured interviews with academics and practitioners from non-government organisations. Participants were selected purposively based on their experience in co-producing research or working within research partnerships. Some participants had worked in humanitarian settings while others had experience co-producing research in non-humanitarian contexts. We used Nvivo to thematically code data.

**Results:**

This paper documents the problems with “research as usual” partnerships in humanitarian settings, specifically: the burden on communities as merely sources of data, certain forms of knowledge being valued over others, lack of reflection on the power hierarchies structuring research partnerships, top-down decision-making and lack of transparency, one-way “capacity-building”, lack of mutual benefit, and rigid research processes and timeframes.

**Conclusion:**

This paper highlights key challenges with standard research practices in humanitarian settings and identifies seven key principles of co-production that can be helpful in attempting to equalise power dynamics within research partnerships, specifically in conflict-affected and humanitarian settings.

**Supplementary Information:**

The online version contains supplementary material available at 10.1186/s13031-021-00399-w.

## Introduction

The notion of “co-production” is increasingly identified as a means of tackling unequal power hierarchies. Originating from the work of Elinor Ostrom [1], co-production emerged out of the recognition that more horizontal partnerships between the public sector and communities were needed to improve delivery of goods and services. Co-production is associated with the idea of creating more participatory processes for the needs of service users to be heard and acted upon. There are many definitions for co-production of services, which vary between disciplines, including: “the process through which inputs used to produce a good or service are contributed by individuals who are not ‘in’ the same organization” [1]; “When you as an individual are involved as an equal partner in designing the support and services you receive” [2]; and “Co-production enables citizens and professionals to share power and work together in equal partnership, to create opportunities for people to access support when they need it and to contribute to social change” [3]. Others have avoided explicitly defining co-production, referring broadly to concepts it is linked to such as “equal partnership and transformation”, and “changing the way public services are conceptualised, designed and delivered” [4]. Some scholars place emphasis on the fact that co-production occurs on a “spectrum”, ranging from consultative to immersive rather than being an outcome that is achieved [5], while others suggest consultation is not co-production [6]. Co-production of public services has often been linked to the health sector, and has been associated with the notion of “public involvement” in decision-making about their health [7, 8].

While co-production has perhaps most often been associated with delivery of public services [9], research and research partnerships have also been identified as needing a co-production approach. A key critique of research is that it does not always have relevance for people most affected, for example communities or service users who are usually not involved in shaping research scope or determining the topic of interest [10]. Academia has been critiqued for policing what counts as knowledge, while devaluing experiential knowledge [11]. Specific to humanitarian settings, scholars have emphasised the problems with top-down “Northern” agency-led research in which “local” actors are marginalised and given tasks by institutions in higher-income contexts [12]. In such research collaborations, the voices of communities most affected by the research are least heard, while outside actors make the decisions and author publications [13]. These power hierarchies can be particularly significant in research conducted within conflict-affected settings, where inequalities are already present [14].

In recent years, what some describe as a co-production “turn” has occurred [15], resulting in wider, even colloquial use of the term across varied disciplines. While some scholars have been swift to distinguish co-production of research from other terms commonly used when describing research partnerships, such as “collaborative” or “participatory” research [9, 16, 17], others draw attention to the value of using other collaborative research models rather than solely relying on the sometimes murky concept of co-production [18] Within the literature, there are varied perspectives on who can be involved in co-producing research, especially on the role of communities themselves [19, 20]. Some literature on co-production highlights that co-producing research requires something more than merely the presence of a partnership. For example, Bell & Pahl suggest that co-production “destabilizes” the “privileged” space of knowledge production that academia has occupied, drawing attention to the fact that co-production enables other kinds of knowledge to be valued [21]. Critical to the notion of co-producing research is the practice of shifting power [15]. While acknowledging the level of commitment, complexities in shifting power, methodological rigour concerns and financial and time investment required to co-produce research [16, 22, 23], there is recognition that co-production can result in improved outcomes through the process of bringing different groups together to share their ideas and expertise, including better health outcomes [24, 25].

While there is burgeoning literature on co-production, we observe that most of it focuses on co-production within service delivery or healthcare settings. While there is some literature on co-production in international development [26–29], there is a paucity of literature on the co-production of research in humanitarian settings. Our study sought to generate evidence on the realities, challenges and benefits of co-producing research in humanitarian settings. It resulted in a Practice Guide designed to help academics and NGO practitioners in co-producing research [30].

The aim of this paper is to draw attention to the main challenges associated with a “research as usual” approach to research partnerships in humanitarian settings. With increasing focus in the humanitarian sector on “localisation” and “decolonisation”, it is becoming more important to reflect on power dynamics within the research process. We propose seven key principles of co-production to address these challenges, drawing on literature as well as semi-structured interviews with academics and practitioners with experience in co-producing research and/or collaborative research partnerships.

## Methods

Our research employed a qualitative methodology. We began by undertaking a comprehensive literature review. This involved searching a range of academic databases (PsycINFO, Social Policy & Practice, Academic Search Complete, Web of Science, Scopus) for “co-production” in the title. Google and Google Scholar were also searched for “co-production” AND “research”. For both the academic databases and Google/Google Scholar, the first 60 results were screened and articles that referenced research, capacity-building or service delivery were read. We also reviewed the reference lists of included articles to identify any additional literature of relevance. Between both authors, we took detailed notes on 128 articles and coded our notes using Nvivo based on key themes in the literature which we identified iteratively.

We used purposive sampling to identify individuals who had co-produced research, and/or who had been involved in some form of academic-NGO-community research partnership. We prioritised interviewing people with experience conducting research in humanitarian settings, however also included individuals with research experience in non-humanitarian contexts due to limited individuals being identified. We aimed to ensure that participants included a diverse group of female and male academics and practitioners from different geographical locations (as outlined in the table below). In total we interviewed 32 participants, 18 of whom were women and 14 were men. Overall, 15 were working as academics, 12 were NGO practitioners, and five were independent researchers or who worked for consultancy firms or (non-academic) research institutes. Some had held different professional roles in the past (for example, academics who had worked for NGOs and vice versa), and thus were able to reflect on multiple positionalities during the interviews. While the table below outlines the geographical location of the individual’s current institution, the individuals themselves did not necessarily hold the same geographical identity as their institutions (Table 1).

**Table 1:** Research participants

Number of interview participants	Europe	North America	Africa	Middle East	Asia	Australia
Academic	9	2	1	2	1	0
International NGO	1	0	1	0	0	0
Local/national NGO	1	0	5	2	2	0
Other (research institutes, social enterprises, independent researchers)	2	1	1	0	0	1

We used topic guides to explore key themes related to: how co-production is defined, the motivations for co-producing research, challenges and benefits of co-producing research, power hierarchies within research and approaches to strengthen capacity. Our topic guide is included in Additional file 1. We conducted interviews using Zoom or Skype. The interviews were audio-recorded and transcribed by a transcription company. The data was coded inductively based on key themes, using Nvivo. All interviews were jointly coded to ensure consistent coding practices and identification of any divergent or additional codes.

The interviews and analysis process were informed by both authors’ experience conducting research and working in humanitarian settings. While neither of us had co-produced research, we were able to understand the challenges, barriers and benefits of co-production for humanitarian settings, based on our previous work experience.

Ethical approval to conduct the interviews was received from the London School of Hygiene & Tropical Medicine (reference: 21789 on 1st May 2020). All respondents provided informed consent and their interview data (specifically quotations included in this article) was anonymised.

## Results

This section outlines the findings on the key challenges associated with “traditional” research approaches, or what could be termed “research as usual”. While the focus is on humanitarian settings, we also include reflections from the participants who conduct research in non-humanitarian settings. We draw attention to seven key themes: burden on communities as merely sources of data, certain forms of knowledge being valued over others, lack of reflection on the power hierarchies structuring research partnerships, top-down decision-making and lack of transparency, one-way “capacity-building”, lack of mutual benefit, and rigid research processes and timeframes. Within each theme we highlight the contrast between research as usual and practitioner/academic experiences of co-produced research.

### Burden on communities as merely sources of data

Many participants reflected on the burden placed on those who are asked to participate in research studies. Of particular relevance for humanitarian settings was the reflection from one academic about “research fatigue” experienced by refugee communities: “People are very fatigued from being asked these same questions by researchers. The same old stuff with no reciprocity, no payback (...) nothing comes out of it ever” (Academic, female, North America). Another academic described how communities might feel: “They wheel in, they take some information from me, and they never come back. They’re not interested in what I have to say or in me having a part to play. They just want the data and that’s it” (Academic, female, Europe). In this framing, communities are repositories of data - a perception that can be difficult to overcome. She added, “The research sometimes has a bad reputation of using people as participants and gathering the information they want, and then just dropping them. I had to do a lot of work to get people to understand that actually, this was a longer-term thing and we wanted to work together.”

Practitioners and academics also reflected on the fact that research findings are not always shared back with communities who participated in the research. “If they cannot get access to it and if they don’t have opportunity to listen to you at the end, I don’t think the cycle is complete” (Academic-practitioner, male, Africa). A male practitioner from Asia reflected on the pressure placed on communities in humanitarian settings: ‘In the height of humanitarian emergencies, people are being assessed to death with so many repetitive assessments’ (NGO, male, Asia).

Co-production was discussed by many as a way of centering the needs of communities: ‘[T]aking the idea of, ‘What does the community actually need?’ takes longer but can be a lot more transformative in the long run” (NGO, female, Africa).

### Certain forms of knowledge being valued over others

During reflections about the way communities are treated during the data collection process, participants also reflected on how certain kinds of knowledge are positioned over others within research: “[I]n a traditional setting, often, the researcher is set up on that pedestal as having the more important knowledge...” (Academic, female, Europe). A practitioner working in an NGO in Africa also challenged the perception that researchers hold all the knowledge: “[T]hey’ve not been on the ground. They know the theories [but] may not be on the ground” (NGO, female, Africa).

In contrast, co-production was identified as a process enabling knowledge to be shared. One NGO practitioner described the process of co-production like this: “I know, you know, and we put our knowledge together and what we don’t know all of us, we go and search it all together. That’s what makes it [co-production] particular” (NGO, male, Africa). An academic also commented, “Co-production is an effort to try to move past that; a recognition that people shouldn’t just be interviewed or subjects of analysis, but should actively be taking part in producing it” (Academic, male, Africa). In this framing, communities move beyond solely being providers of data, as outlined in the section above, but are involved in producing knowledge themselves. One academic put it like this:

They [refugee communities] have to be part of the process from the beginning, from conceptualizing the project, to the final evaluation of the project. They have to have a place on the table and if we're doing research on these NGOs and on the services we're providing to the NGOs and on refugees or on communities, they have to sit, have a place at the table and they have to participate. We have to be very transparent with them. I think some organizations do this, but they are rare, very few. Although it's written in their code of ethics. Although they write it in their project documents… (Academic, female, Middle East)

### Lack of reflection on the power hierarchies structuring research partnerships

The topic that was perhaps most discussed was the power hierarchies present within research partnerships. While much of the discussions focused on power disparities between researchers and communities, others drew attention to power dynamics between academics and NGOs, and between “Northern” and “Southern” institutions.

One practitioner from Europe emphasised the need for individuals within research partnerships to reflect on and identify the power hierarchies they are situated within and address them: “I don’t think you can really collaborate well unless you acknowledge what people step into the room with in terms of power and what you would like them to step out of the room with and how the processes need to change a little bit to accommodate that.” (NGO, female, Europe). For her, the research process itself needed to adapt to challenge power hierarchies.

One male academic articulated his own power, while acknowledging that addressing unequal power can be difficult when there are established institutional hierarchies that people are used to operating within:

[I]n my experience... I am the power. I really try and work hard to listen to the voices of the less powerful within the organization and we try and promote mechanisms that will give voice to the underrepresented, to the early career researchers, to the women, to make sure that we respect the cultures of the communities that we're working in. I have no doubt that no matter how I try, people's attitudes change when I come into the meeting because I'm perceived as the person with the final say, as the principal investigator. I have no doubt about that and it means that no matter how much we try to level, to promote the ability of people to influence the decisions and to make decisions, ultimately there's an awful lot of deference to authority (Academic, male, Europe).

A female academic echoed this challenge: “Even if they’re trying not to make decisions, even if they’re trying to be egalitarian, they’re perceived by others to have the power. People defer to them or people think they can’t argue back or point out their mistakes because there’s a perception of this power that is hard to overcome. That’s a big part of the power pieces that need to be talked about” (Academic, female, USA).

Practitioners and academics identified the importance of reflecting on power hierarchies when co-producing research, noting aspects like interrogating our own processes (Academic, male, North America) and recognising that while some people are dynamic leaders, others will want to contribute in different ways, and should be supported in doing so (Female, NGO, Europe).

One participant emphasised the structure and operations of the humanitarian industry that determine “whose voice matters and whose voice doesn’t matter”. He observed: “I think being able to show the issues that we often are concerned within humanitarian settings, the access to basic rights and needs, and those things, cannot be divorced from these broader structural issues that involve the role and the system of humanitarian organizations” (Male, academic, North America).

These wider structural issues in humanitarian settings also echo literature stating that co-production may be aspirational and incremental rather than being an outcome that is achieved (Carter et al., 2019). One academic reflected that co-production is a “guiding light” rather than a concrete achievement (Academic, female, Europe). A practitioner in Africa highlighted the importance of identifying when and what can be co-produced:

Choosing when to do co-production is actually quite important I think when you don't have the luxury of doing everything in a co-produced way. You have to pick your moments about what parts are going to be fully co-produced and what parts were responsibilities and people go and do those parts on their own (NGO, female, Africa).

### Top-down decision-making and lack of transparency

In articulating the complex, multi-layered power hierarchies that structure research partnerships, especially in humanitarian settings, practitioners and academics also discussed how research partnerships may be characterised by top-down decision-making and poor transparency. One practitioner from Asia described a situation they face: “[A] partner ... [has] a ... cookie-cut prescribed product that they want. ... They’ll ask about the structure of the data collection format. They wouldn’t give us the reason behind it, they wouldn’t bother about the philosophy of it. ‘Just give me the data and I will train you how to administer the instrument.’” (Male, NGO practitioner, Asia).

Others highlighted how the structure of research funding embeds unequal dynamics into partnerships because of the requirement that a certain (often Northern-based) institution be lead researcher. This also influences how research resources are shared: “Community partners and practice-based partners traditionally get a pittance really for the time they put in.” (Social enterprise, female, Europe).

Practitioners and academics emphasised the importance of creating clear governance structures for decision-making: “Because no actor should be the ultimate decision maker on how to allocate resources, there should be [a] different governance structure (...) [T]hey need to decide collaboratively on how for example, resources are allocated or how different decisions can be made.” (Academic, male, Europe).

Across many interviews, there was strong focus on co-production requiring that research partners are involved from the outset of the research process: “I think the key in these is how you involve these partners, and at which stage. Do you actually involve them since the beginning, or you just call, ‘Hey, I have a grant for research, we think that we’re a good partner and here it is. We already agreed on everything with the donor, and we just have to be implementing.’” (NGO, male, Middle East).

Others drew attention to the practicalities of ensuring equity within decision-making within co-produced research:

I think most people just don't even think about that when they're starting those processes. They think, ‘Oh, well, I'll convene a meeting and it's open to everyone.’ There, it's open, but it might be at a time that some people have caring responsibilities or are at work, it might be in a place that you can't get to with public transport, it might be some people can't afford to get there on public transport, all of those dynamics (Social enterprise, female, Europe).

### One-way ‘capacity-building’

During interviews with practitioners and academics, the topic of capacity-building was identified as an area where top-down and North-South interactions might be reinforced within research partnerships. One participant discussed the “‘political economy of knowledge production” between Western and Southern universities. He explained, “Most of the researchers in [West Africa] for instance, were not very comfortable with the use of the term that the western university is going to strengthen the capacity of southern university” (Academic, male, Africa).

While nearly all participants emphasised the importance of capacity strengthening when co-producing research, there were a range of views about what constitutes capacity strengthening and what it means in the context of co-produced research. While some participants from local NGOs identified a need for capacity strengthening, particularly around technical skills, others problematised the concept: “I think particularly at the moment there’s quite a lot of rejection of the term capacity building…It suggests an imbalance of capacity that many local organizations would reject” (Academic, male, Europe).

Yet even those who challenged the concept of *one-way* capacity strengthening recognised the significant benefits of a mutual exchange of information, skills and capacity. NGOs were recognised as having significant contextual knowledge and experience designing and undertaking operational research that they could share with academics. One participant gave examples of how when graduate students from an ivy league university came to co-produce research, the NGO he worked for in an African country benefited from the experiences, and strengthened the students’ capacity around issues such as gender mainstreaming, local systems of governance, security, logistics, and communication. Another female academic from Europe said:

I think as researchers, if we're doing co-production, we need to think about having our own capacity strengthened, not about how we might strengthen the capacity of communities or individuals or participants, because again, that's the paternalistic model that I really would like to see us move away from. (Female, academic, Europe)

### Lack of mutual benefit

Practitioners and academics discussed the challenges they face when research only benefits “If we really want equitable partnership, there has to be an incentive from both sides to do that.” (Academic, male, Africa). A practitioner from Africa commented:

A lot of the time, we expect participants to just give their time for free. We also expect people to be part of something that then has an academic paper as an outcome. That has very little relevance to most of the stakeholders that you're engaging with. There's got to be something in it for them. There's got to be a stake in it for them. There's got to be some value in them attending (NGO, female, Africa).

Participants stressed the importance of all stakeholders in co-production feeling like they are benefiting from their involvement: “Everybody who’s involved has respect for diversity of experience and perspective, we always make sure there’s mutual benefit for everyone, and we’re always checking and challenging each other throughout.” (Academic, female, Europe). A practitioner in Africa also drew attention to the economic imbalances that need to be considered when co-producing research:

I think recognizing that you can't just expect people to aimlessly make time for co-production. They've got to see a value in it. It doesn't have to be a financial value, but sometimes that is a helpful way of dealing with the realities of the situation. In some cases, it was like a fisherman would have to miss a day's catch to be part of the co-production process. Is it worth it for him economically? Can he afford to give up a day's work? Those are things I think people in the research community need to take a little bit more into consideration (NGO, female, Africa).

Some positioned co-production as different from other research approaches (particularly those that may seem extractive to participants), in that a core tenant of co-production is ensuring everyone gets something from it: “[T]here’s this principle of reciprocity. It’s mutual, it’s not one way traffic (Female, academic, Europe). For some participants, this meant paying collaborators/participants, either in cash or in kind. While there are divergent views regarding the ethical imperatives and challenges of paying research participants in humanitarian settings, participants emphasised that reciprocity does not necessarily involve payment – it is about a mutual exchange of time, knowledge, capacity, resources and ultimately value.

The potentially divergent incentives for academics and non-academics can pose challenges when they work together to co-produce research – ensuring reciprocity is emphasised throughout is one way to mitigate this.

### Rigid research processes and timeframes

Practitioners and academics discussed how rigid processes and timeframes can reinforce power hierarchies within research partnerships. The requirement from academic institutions that local ethics approval be sought was described as challenging by many practitioners, with one stating that approvals were a “headache”: “You easily get them there in the north, but here, you may easily spend three months looking for ethic authorization. Either the person to give you that does not understand what you’re talking about, either the person is absent, either they want you to pay money, either they want you to bring the project and then you bring the project and they don’t get time to read it. That is a nightmare.” (NGO, male, Africa). Others discussed the challenge to produce research in a timely manner within NGO-academia research partnerships. One academic talked about the “unpredictability” and lack of control that might occur within research partnerships, explaining that this is why many choose easier options: “There’s an incentive to quick, dirty, high-profile projects” (Academic, male, Africa).

In exploring the benefits of co-production, participants emphasised the importance of flexibility, while also acknowledging that flexibility required trade-offs. These include academics (and others in positions of power) surrendering control to accommodate others; working without a rigid plan or timeline for publications; and the potential use of extra resources (time, money). Respondents also noted the importance of donors taking a flexible approach to the co-produced research they fund. As a practitioner said when discussing flexibility as a principle of co-production:

I think projects tend to suffer when donors require very stringent outputs at the outset. They say, ‘Oh, take a co-production approach, but you need to produce these results.’ [chuckles] “It doesn't give you much ability to actually use the co-production process for the benefit that it could have. Funders need to be a lot more flexible about co-produced projects and let the outcomes emerge (NGO, female, Africa).

## Discussion

Our study identified key challenges within ‘research as usual’, specifically the burden on communities as merely sources of data, certain forms of knowledge being valued over others, lack of reflection on the power hierarchies structuring research partnerships, top-down decision-making and lack of transparency, one-way ‘capacity-building’, lack of mutual benefit, and rigid research processes and timeframes. These challenges become particularly accentuated in humanitarian settings. With the increased focus on decolonising and localising the humanitarian response, attention has also been placed on how research processes might need to shift. Our findings resonate with existing literature on co-production, however it is important to note that this literature is largely outside the humanitarian field since most co-production has occurred outside of humanitarian settings.

Participants in our study observed the challenges for research when communities are merely sources of information rather than having a stake in research conducted about them. Within literature on co-production and participation in service delivery, scholars have pointed to the need for service users to be involved in knowledge produced about them [2, 31], while acknowledging the challenges associated with this level of engagement [19, 20]. In humanitarian settings, research may be extractive, focused on obtaining data from communities in the most efficient way possible rather than through participatory processes that are grounded in the lived experiences of conflict-affected communities [32]. Across the literature on co-production in both non-humanitarian and humanitarian settings [11, 13, 33–35], scholars emphasise what we found in our study, that expert or international knowledge is often valued over local or contextual knowledge. In humanitarian settings in particular, the notion of an outsider coming into a humanitarian emergency to provide guidance continues to persist, marginalising the voices of those who already know the setting [12, 13].

Our findings revealed multiple levels of power hierarchies that result in academic knowledge being valued over other kinds of knowledge, such as knowledge held by communities and knowledge of local actors. Across all the interviews, the topic of power dynamics within research partnerships generated significant discussion. Participants were concerned about the lack of critical reflection about power hierarchies that they sometimes witnessed within research partnerships, while emphasising their own efforts at being more reflexive in thinking about the power they held. This finding echoes literature that suggests the first step to addressing unequal power is to make the hierarchies visible [11]. Research partnerships, especially those in humanitarian settings, do not always intentionally involve critical reflection on power hierarchies, however this reflection lays the groundwork for conversations about shifting power intentionally at every step of the research process [8].

A key way power manifests itself is through decision-making. Participants noted the importance of co-produced research being relevant to local contexts and decision-making processes in humanitarian settings, rather than being purely theoretical or abstract. Both the literature and participants highlighted the importance of stakeholders involved in co-producing research making decisions equitably, collaboratively or jointly, and noted this is often not the case in traditional research. While some participants and literature described making decisions equally and through consensus [36], others emphasised that decision-making should be equitable rather than equal, and may involve trusting the stakeholders who are best informed to make decisions to do so [37] rather than involving everyone in every decision [38]. Participants reflected on the need for ordinarily more powerful voices to be excluded from some decision-making processes and spaces, as part of levelling the playing field, for example, asking those in positions of power to speak last during decision-making processes, so that others have opportunities to voice their opinions.

The literature highlights the importance of embedding practices of mutual capacity-strengthening into research, and identifies the benefits that can be realised when multiple actors pool their differing knowledge, insights and expertise into the research partnership [25]. Capacity strengthening was also recognised by participants and in the literature as a means through which to consider and redress power imbalances when co-producing research [39]. While in the literature capacity is often framed in conceptual terms [40], participants spoke of capacity strengthening in more practical terms. This is not surprising, as when co-producing research it is helpful for discussions about capacity to be grounded in the research process. The importance of capacity strengthening being mutual rather than one way, as often exists in development and humanitarian contexts [40], was emphasised by participants.

Similarly, flexibility was consistently mentioned in the literature and by research participants as an essential element of co-producing research. This includes flexibility in partnerships and approaches [41], methodology [42], and flexibility within roles [43]. Participants recognised that rigid, traditional research approaches are challenging to implement in partnerships focused on humanitarian issues, and that even more flexibility than usual is required when co-producing research in humanitarian settings, as the situation on the ground (including needs of affected populations, access, and security) can change rapidly.

**Fig. 1:**
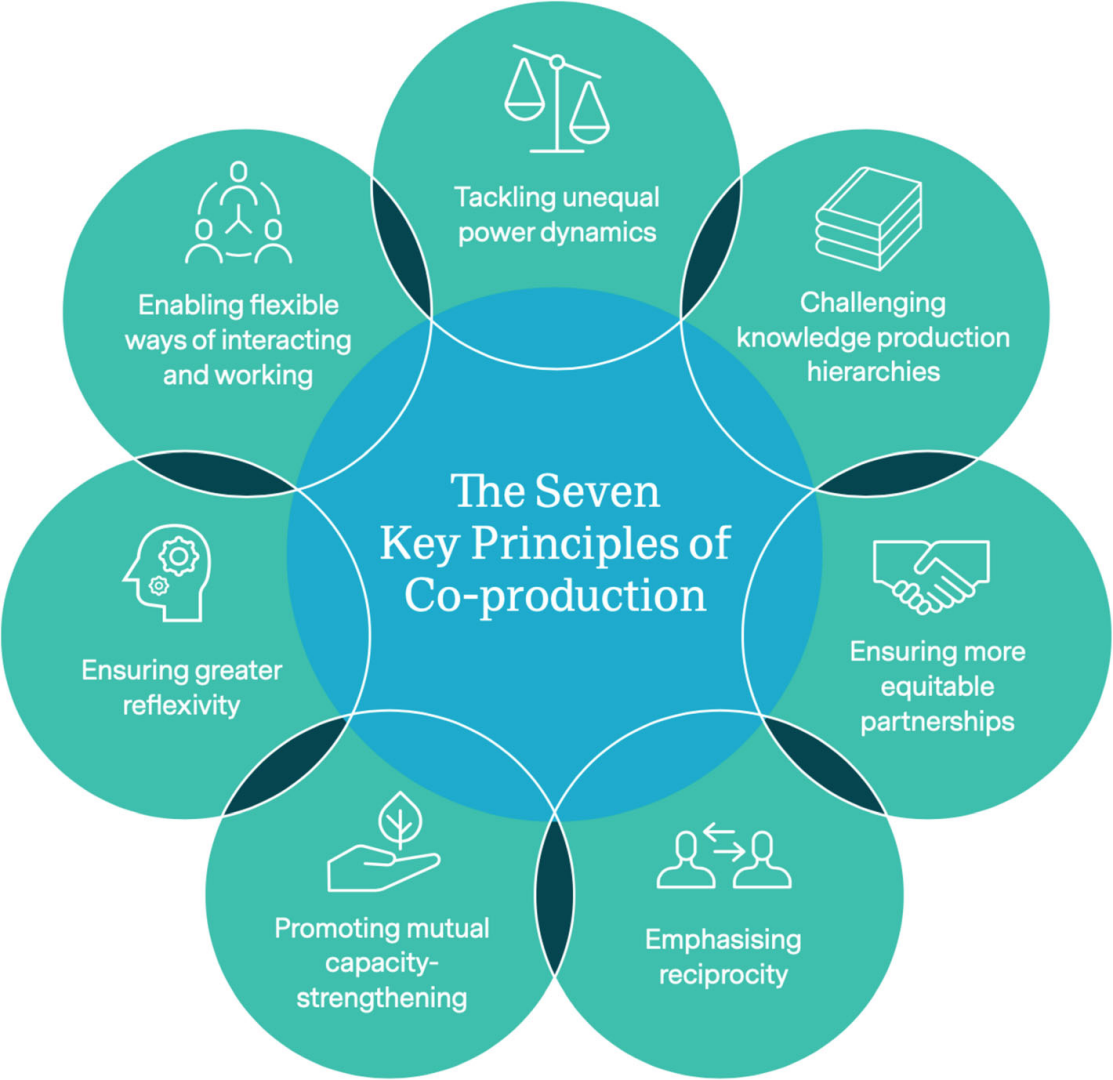
The seven key principles of co-production

Reproduced with permission from [30].

As the above discussion suggests, there was significant overlap between key issues regarding co-production that are discussed in the literature and those raised by participants in our research. Our research generated unique insight, however, into the issues and principles of co-producing research in humanitarian settings. Due to the paucity of literature on co-production in humanitarian settings, the unique contribution of this paper is strengthening knowledge about the value of research co-production for humanitarian settings. We have previously articulated a definition of co-production within research [30] as: “a horizontal partnership between researchers (both academic and non-academic) and active research participants to undertake research that can inform action.” Our definition differs from existing literature in focusing on research rather than service provision and includes seven key principles of co-production, which as the findings in this paper suggest, help to address each of the challenges associated with traditional research. These seven principles, which we ground in existing literature on co-production, draw attention to the power hierarchies underlying research processes, pointing to strategies that can be used to equalise power dynamics when co-producing research in humanitarian settings (Fig. 1).

### Tackling unequal power dynamics

Co-production requires explicitly examining and shifting who holds power and how they use it throughout the research cycle. Our study identified the importance of critical reflection on power hierarchies throughout the research process. For co-produced research on humanitarian issues, this includes identifying who has power over allocating budget and resources, reflecting on how the research topic is identified, and understanding the power dynamics underlying the fieldwork process, among others. Our findings build on existing literature that suggests shifting power “involves unlearning well-established practices” [44], a process that may be messy and difficult, but is essential for the realisation of other co-production principles discussed below. As Darby aptly writes, “Attempting co-production requires engaging in messy processes of negotiating power structures and diverse values, confronting our academic positionality, and risking letting go of control of outcomes (and outputs) of research” [45]. Interrogating power imbalances and exploring how power can be more equitably distributed between collaborators is central to the work of co-production.

### Challenging knowledge production hierarchies

Our study highlights that co-producing research in humanitarian settings requires academics and practitioners to shift from their roles as experts and implementers to collaborators who value experiential as well as academic knowledge. Participants emphasised that people affected by crisis who are involved in the research should be recognised not only as data sources, but as the experts of their own experience and communities. As Beebeejaun et al., write, “Co-produced research inherently re-conceptualizes the role of the researcher in working with communities, as more accepting of different claims to knowledge, operating within new shared spaces for acting, committed to social change, and perhaps, willing to trade-off the ‘traditional’ forms of academic reward for community benefit” [46]. Challenging whose knowledge is valued includes creating space for the voices of Global South researchers and organisations, which is part of decolonising humanitarian aid [34, 35, 47].

### Ensuring more equitable partnerships and shared decision-making

Creating equitable partnerships and sharing decision-making is at the heart of co-production. While scholars have identified *equality* as being a central component of co-production [6, 8], we distinguish between equality and equity, and choose to emphasise the latter. Our participants highlighted that while power and capacity to participate should be equal between collaborators, collaborators’ contributions will vary according to their unique capacities and resources. While the division of work may not be equal, however on balance it should be fair, equitable and mutually agreed. In order for partnerships to be equitable and decision-making to be shared, it is important to facilitate inclusion and participation [48]; as Clarke et al. write: “The task of establishing inclusivity is not something that is ever completed, as inclusivity is continuously negotiated through every day and mundane situated practices. These encounters add up over time, creating expectations, setting the ‘order’ of how the group operates, and providing members with implicit social guidelines for what can be discussed, questioned, and actioned” [49]. Our study participants identified the importance of each actor being involved from the outset in co-produced research, to set the tone for future equitable decision-making processes. They also discussed the importance of addressing practical barriers to equitable decision-making.

### Emphasising reciprocity

In our study, participants discussed the importance of each actor in the co-produced research obtaining some kind of benefit. Successful co-production involves reciprocity [4] - give and take or mutual exchange of time, skills, and knowledge. It is particularly important because while the incentives of collaborators involved in co-production are likely to differ [50], it is very important that all stakeholders feel they are not only giving, but getting something out of the process. Clark describes reciprocity as something that “builds trust, connections and mutual respect between people” [2] all of which go a long way towards facilitating a positive and productive co-production experience.

### Promoting mutual capacity strengthening

Promoting mutual exchange of knowledge, skills and capacity is a key aspect of co-producing research. Participants discussed the importance of mutual rather than one-way exchanges that position certain actors as experts and others whose capacity needs to be built. The focus on mutual capacity strengthening necessitates a change from historical and current forms of capacity strengthening in the humanitarian and development sectors, which tend to be one-way, with ‘Northern’ institutions endeavouring to strengthen the capacity of local individuals and NGOs [39, 40]. As a starting point, this requires valuing different types of knowledge (such as experiential and local knowledge) rather than privileging academic expertise, and being willing to learn from all collaborators. As Sibai at al. write, “trust regional research capacity and contribution...There is much to learn from regional scholars—as insiders on the refugee crisis who have long experienced the systems of governance under study. They speak the language, have rich empirical experience, know the sensitivities and how to navigate them, and can provide contextualised insights into the findings” [12].

### Ensuring greater reflexivity

Participants in this study identified reflexivity as key to challenging prescribed roles and shifting power in research partnerships. We define reflexivity in research as the processing of “critically reflecting on all aspects of the partnership and research cycle, specifically thinking about how our positionality (our own background, culture, identity) and perspectives (assumptions, beliefs, worldviews) shape the research process” [30]. Reflexivity opens up space to consider not only our individual positionality and perspectives, but the broader systemic positions, perspectives and power dynamics that affect how both research and practice happen in humanitarian settings [51]. Rose & Kalathil write: “Until we are able to actively reflect on our own entrenched positions of privilege, and how the established history of ideas perpetuate that privilege, co-production will fail in its stated aim of democratizing knowledge production” [11].

### Enabling flexible ways of interacting and working

As the process of co-production involves learning and evolving, co-producing research requires flexibility from stakeholders in how they work and interact [52]. Participants emphasised the importance of being willing to surrender control and adopt more flexible, but perhaps more time-intensive ways of working while co-producing research. According to existing research, being flexible with roles and ways of working while co-producing research can help stakeholders to build trust [43]; accommodate uncertainty [41]; be responsive to ‘real-world’ demands [53]; and enable collaborators to soften and strengthen boundaries with each other as and when appropriate [54]. As Pain et al., write in their discussion on co-production, and specifically on the relationship between research and impact, “flexibility is a vital operating principle” [25]. Part of changing how research is done might involve jointly writing up research findings, so that those who collected the data or were research participants are also given opportunities to participate in analysis and writing. As others have noted, joint writing can be challenging and requires greater time commitment and flexibility, however the process of working alongside communities to analyse data about them brings significant depth to research findings [55–57].

### Limitations

Importantly, while the subject of the research was co-production, our study was not itself co-produced. We did not interview community members themselves due to limited resources and time to conduct interviews, which means that the reflections on co-production are solely from the perspective of practitioners and academics. However, these practitioners and academics did reflect on how communities are involved (or not) within research partnerships and co-production, and the interviews involved reflection on power dynamics with communities. Due to COVID-19, it was difficult to interview some practitioners, who were often involved in coordinating responses to the pandemic. It was also challenging to identify people to interview who had first-hand experience co-producing research in humanitarian settings. We therefore included some participants who had significant experience co-producing research in non-humanitarian settings, as they were able to reflect on fundamental principles, challenges and benefits that are relevant to conducting co-produced research in any setting. We interviewed more participants than initially planned, in order to ensure we had geographical diversity among the participants, however many participants were European academics.

## Conclusion

We suggest that the challenges associated with research as usual discussed in this paper are far too significant to ignore, and that even an incremental approach to co-producing research that seeks to shift power where possible, is an important starting-point [5]. While co-production may be challenging, practitioners and academics who participated in our research identified co-production as a way of addressing the power hierarchies structuring research partnerships. They recognised that co-production is not always a straight-forward process, that entrenched power hierarchies structuring research and humanitarian aid are difficult to unravel, and that co-production is a process rather than a concrete outcome. The seven principles outlined in this paper each correspond to the challenges associated with “research as usual”. We suggest these principles represent an important starting-point for practitioners and academics who are committed to equalising power within research partnerships.

## Supplementary Information

**Additional file 1.** Semi-structured interview guide.

## Data Availability

The interview transcripts generated during this study are not publicly available since participants did not give consent for the public sharing of their information. We have made the topic guide for the interviews available as a supplementary file.
