# Supplementary material for: Research as usual in humanitarian settings? Equalising power in academic-NGO research partnerships through co-production
Source: Confl Health. 2021 Aug 26;15:64. doi: 10.1186/s13031-021-00399-w (PMC8390113; doi:10.1186/s13031-021-00399-w)
Supplement: Supplementary file 1 — Additional file 1. Semi-structured interview guide. [file 13031_2021_399_MOESM1_ESM.docx]

**Annex 1: Key informant interview guide**

1. What does co-production (or collaboration – if participant hasn’t explicitly worked on co-production) during research mean to you? What do you think are the key principles of co-production?
2. What motivates you/your organisation/other groups in general to co-produce research? In what circumstances do people decide to co-produce research?
3. If they explicitly work on co-production: In what ways may co-production differ from other terms we use like ‘collaboration’, co-design or ‘participation’ - where are the similarities/differences with co-production?
4. Can you share any examples of co-production (or collaboration) that you’ve seen or participated in? Who was involved in this co-production example? What was co-produced?
5. For academics only: In your experience, what do you think are the main challenges academics face in co-producing research with NGOs and communities? Prompt: can you share any examples? (academic mindset – how it translates)
6. For INGOs only: In your experience, what do you think are the main challenges international NGOs face in co-producing research with academics and communities? Prompt: can you share any examples?
7. For LNGOs only: In your experience, what do you think are the main challenges local NGOs or community-based organisations face in co-producing research with other actors, including international NGOs, academics and communities? Prompt: can you share any examples?
8. What unique challenges or barriers might be faced when co-producing research within humanitarian settings – including refugee camps or protracted emergencies? Follow-up: How might these challenges be addressed?
9. How do power hierarchies, e.g. power hierarchies related to gender, race, ethnicity, influence the co-production process?
10. ‘Capacity-strengthening’ or ‘capacity-building’ are terms we often use when working with different groups of stakeholders to co-produce research. Can you share examples of where you felt capacity-strengthening/building has worked well when co-producing research? Can you share any examples of where it has not worked well? Prompt: Why?
11. In what ways might compromises be needed when co-producing research? Can you share examples of different individuals/institutes making compromises for the sake of the relationship?
12. What strategies can be used to ensure that service users/research participants are also involved in co-production? Prompt: Can you share examples of where this occurred successfully? What are the barriers to this?
13. What advice would you offer to anyone who is interested in co-producing research?
14. Is there anything you’d like to add? Questions?
